# Long-Term Effect of an Aqueous *Fraxinus excelsior* L. Seed Extract in Spontaneously Hypertensive Rats

**DOI:** 10.1155/2014/565212

**Published:** 2014-02-18

**Authors:** Noemi López-Carreras, Sandra Fernández-Vallinas, Marta Miguel, Amaya Aleixandre

**Affiliations:** ^1^Departmento de Farmacología, Facultad de Medicina, Universidad de Complutense, Avenida Complutense s/n, 28040 Madrid, Spain; ^2^Instituto de Investigación en Ciencias de Alimentación (CIAL) (CSIC-UAM), C/Nicolás Cabrera, 9, 28049 Madrid, Spain

## Abstract

The effect of long-term intake of different doses (20, 40, and 60 mg/kg/day) of a *Fraxinus excelsior* L. seed extract (FESE) on spontaneously hypertensive rats (SHR) was evaluated. Water was used as control and captopril (50 mg/kg/day) was used as positive control. Systolic blood pressure, body weight, and food and liquid intake were registered weekly in SHR. The antioxidant and vascular relaxing properties of FESE were also studied in these animals. The development of hypertension was attenuated in the groups treated with captopril or FESE. The antihypertensive effect was more accentuated in the captopril group than in the FESE groups, and it was paradoxically more accentuated in the groups treated with 20 mg/kg/day or 40 mg/kg/day of FESE than in the group treated with the highest dose of this extract. Body weight gain and food intake increased in the FESE groups. After removing the corresponding antihypertensive treatment, the arterial blood pressure and the body weight of the FESE treated animals returned to control values. In addition, FESE increased plasma antioxidant capacity and decreased plasma and liver malondialdehyde levels. Moreover, acetylcholine relaxation improved in the aorta rings from the FESE treated rats.

## 1. Introduction

Arterial hypertension is one of the most common causes of cardio- and cerebrovascular complications. Although many effective chemically synthesized drugs exist for the treatment of hypertension, there is a great deal of interest in using natural plant extracts to attenuate the increased blood pressure levels in hypertensive patients and to prevent the cardiovascular disorders associated with this disease [1–3].

The beneficial properties of many plants have been frequently attributed to the presence of phenolic compounds. Among them, iridoids, a particularly widespread group of monoterpenes, retain their chemical composition even when exposed to stressful environments or after processing. Therefore, iridoids may be considered more stable and more bioavailable than other well-known phenolic compounds, as flavonoids [4]. In addition, several biological properties have been attributed to iridoids which have been recognized as important components of medicine and healthy diet [5, 6]. Despite the many known biological properties of these compounds, their effects on blood pressure have been poorly investigated [7–9].

A *Fraxinus excelsior* L. seed extract (FESE) was obtained via a patented process [10]. The analytical study of FESE revealed that it was rich in nuzhenide and GI3, two phenolic compounds belonging to the secoiridoid type [11]. FESE lowered the incremental postprandial plasma glucose concentration in nondiabetic volunteers [11] and limited weight gain and hyperglycemia in high-fat induced obese mice [12]. Recently, we have demonstrated the short-term antihypertensive effect of this extract in spontaneously hypertensive rats (SHR), a model for essential hypertension in humans. FESE also showed diuretic and antioxidant properties that could contribute to the decreased arterial blood pressure in the SHR [13]. In addition, the safety and tolerability of this extract for consumption in healthy subjects have also been demonstrated [14].

Hypertension is a chronic disease that needs chronic treatment. According to this idea, the aim of this study was to evaluate the effect of the long-term intake of FESE on the development of hypertension of SHR. Weight gain and food and liquid intake were evaluated in the animals throughout the experimental period. We also investigate the possible antioxidant properties of FESE by analysing plasma and liver tissue samples from the different groups of rats. Moreover, we carried out complementary trials in aorta ring preparations obtained from these rats in order to investigate the possible modification of endothelial function by this extract.

## 2. Material and Methods

### 2.1. General Protocol in the Rats and Blood Pressure Measurements

After being weaned at 3 weeks, 60 male SHR (Charles River Laboratories España S.A.) were caged in groups of five rats at a temperature of 23°C with 12 h light/dark cycles. They were in turn randomly divided into six groups of 10 animals with *ad libitum* intake. During the experimental period, the SHR of the established groups were fed on a food standard diet for rats (A04 Panlab, Barcelona, Spain). The rats drank different fluids from weaning until the 20th week of life (treatment period) and received therefore different treatments: tap water (negative control), FESE 20 mg/kg/day, FESE 40 mg/kg/day, FESE 60 mg/kg/day, or captopril (Sigma, USA) 50 mg/kg/day (positive control). The quantities of the different products, to be daily administered during this period of life, were selected according to the results obtained when we established the short-term antihypertensive effect of FESE in SHR [13]. From the 20th to the 24th week of life all the animals were given tap water (follow-up period). Five 20-week-old rats of each group were sacrificed by decapitation. Blood and liver samples were collected in these animals. We measured the antioxidant capacity of the plasma samples and determined malondialdehyde (MDA) plasma levels. Liver MDA concentration was also determined. Moreover, the thoraxes of the sacrificed rats were opened and the aorta was quickly extracted to evaluate the endothelial function in accordance with the studies published by Furchgott in 1980 and in 1999 [15, 16]. All these experiments are described below.

Systolic blood pressure (SBP) was measured weekly in the rats from the 6th to 24th week of life, by the tail cuff method [17]. Before the measurements, the rats were kept at 38°C for 10–15 minutes to make the pulsations of the tail artery detectable. Arterial blood pressure measurements were performed at the same time of day (between 9 a.m. and 13 p.m.) in order to avoid the influence of the circadian cycle, and the values of SBP were obtained by estimating the average reading of five measurements. Moreover, the person who measured the arterial blood pressure in the animals did not know the drinking fluid that had been administered to each group.

The body weight of the rats was recorded weekly up to the 24th week of life. Daily intake of drinking fluids and freely accessible feed was also estimated weekly in the SHR from the different groups throughout the experimental period.

At the end of the experimental period, the 24-week-old rats were sacrificed by decapitation. The same determinations and procedures took place in these animals as for the sacrificed 20-week-old rats.

All the above-mentioned experiments were performed as authorized for scientific research (European Directive 86/609/CEE and Royal Decree 223/1988 of the Spanish Ministry of Agriculture, Fisheries and Food).

### 2.2. Plasma and Tissue Preparations

The blood samples were collected into tubes containing lithium heparin as anticoagulant and centrifuged at 3500 g for 20 min to obtain the plasma. Livers were homogenized at 4°C in a Potter with phosphate buffered saline (PBS) (0.01 M PBS, 0.15 M NaCl, pH 7.4). The homogenates were centrifuged at 5000 g for 15 min at 4°C and the supernatant was recovered. The plasma and the supernatants of the centrifuged samples were kept frozen at 80°C until analysis. The liver protein content was determined by the bicinchoninic acid assay (Pierce, Rockford, Ill, USA), using bovine serum albumin as standard.

### 2.3. Plasma Antioxidant Capacity

We measured the antioxidant capacity of the plasma samples by using the oxygen radical absorbance capacity (ORAC) assay, as previously described in Manso et al. (2008) [18]. Briefly, the final assay mixture contained fluorescein as oxidable substrate, 2,2′-Azobis(2-amidinopropane) dihydrochloride (AAPH) as oxygen radical generator, and the antioxidant trolox either as standard or plasma samples. Samples and standards were dissolved in PBS (pH 7.4). A Polarstar Galaxy plate reader with a 485-P excitation and a 520-P emission filter was used. Black 96-well microplates were used. Fluorescence measurement was carried out at 37°C and was recorded every minute for 80 min. ORAC values were calculated and expressed as *μ*mol of trolox equivalent/mL of plasma.

### 2.4. Measurement of Malondialdehyde

Plasma and liver MDA levels were measured by the thiobarbituric (TBA) assay previously described also in Manso et al. (2008) [18]. Briefly, plasma and homogenates of tissues were mixed with trichloroacetic acid, and the tubes were kept in ice for 20 min. Samples were centrifuged at 1500 g for 15 min. before adding TBA to the supernatant; then, the mixture was boiled at 97°C for 30 min. Spectrophotometric measurements at 535 nm were made at 20°C. Plasma MDA values were expressed as nmol MDA/mL and, for the different tissues, as nmol MDA/mg tissue protein.

### 2.5. Experiments in Aorta Rings

The thoracic aorta was carefully cleaned of fat and connective tissue and cut into rings (approximately 4 mm in length). The aorta rings were mounted between two steel hooks in isolated tissue chambers containing Krebs-Henseleit solution with the following compositions (in mmol/L): NaCl, 118; KCl, 4.7; CaCl_2_, 2.5; KH_2_PO_4_, 1.2; MgSO_4_, 1.2; NaHCO_3_, 25; and glucose, 10.0. The medium was gassed with carbogen and kept at 37°C. All rings were allowed to equilibrate for 1 h at a resting tension of 2 g before adding drugs. Isometric tension was recorded by using an isometric force displacement transducer connected to an acquisition system (Protos 5, Panlab, Spain). After the equilibration period, the rings were first contracted by 80 mM KCl to assess their functionality, and when the contraction had reached the steady state (about 15 min) the preparations were washed until the basal tension was recovered. Then the vessels were exposed to methoxamine (10^−5 ^M), and the presence of functional endothelium was assessed by the ability of acetylcholine (ACh, 10^−9^ to 10^−5 ^M) to induce relaxation. Relaxant responses to ACh were expressed as a percentage of the precontraction induced by methoxamine.

### 2.6. Statistical Analysis

The results are expressed as mean values ± SEM for 5–10 experiments and were analysed by a one- or two-way analysis of variance (ANOVA), by using the GraphPad Prism software. For the analysis, we considered, on the one hand, the treatment period (from weaning until the 20th week of life) and, on the other hand, the follow-up period (from the 20th to the 24th week of life). Differences between the groups were assessed by the Bonferroni test and were considered to be significant when *P* < 0.05.

## 3. Results

### 3.1. Arterial Blood Pressure

**Figure 1:**
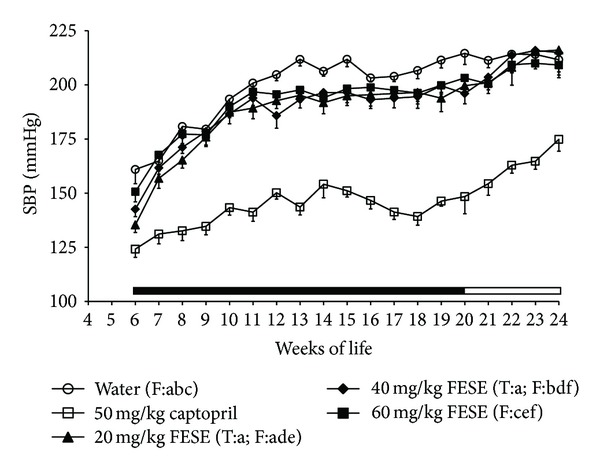
Systolic blood pressure (SBP) of spontaneously hypertensive rats. The animals drank different fluids from weaning until the 20th week of life (treatment period = T, indicated by a solid bar) and received therefore different daily treatments: tap water, 20 mg/kg/day *Fraxinus excelsior* L. seed extract (FESE), 40 mg/kg/day FESE, 60 mg/kg/day FESE, or 50 mg/kg/day captopril. All rats drank tap water from the 20th week of life until the 24th week of life (follow-up period = F, indicated by an open bar). Data are mean values ± SEM for 10 animals in T and for 5 animals in F. Similar letters represent no statistical differences (*P* > 0.05). *P* estimated by two-way ANOVA.

**Figure 2:**
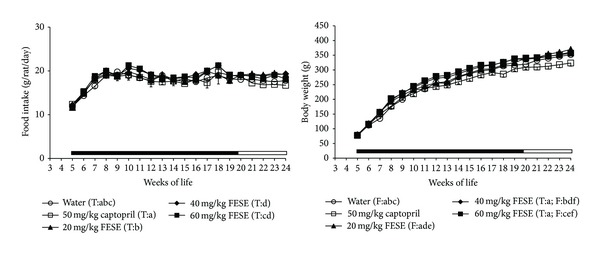
Food intake and body weight of spontaneously hypertensive rats. The animals drank different fluids from weaning until the 20th week of life (treatment period = T, indicated by a solid bar) and received therefore different daily treatments: tap water, 20 mg/kg/day *Fraxinus excelsior* L. seed extract (FESE), 40 mg/kg/day FESE, 60 mg/kg/day FESE, or 50 mg/kg/day captopril. All rats drank tap water from the 20th week of life until the 24th week of life (follow-up period = F, indicated by an open bar). Data are mean values ± SEM for 10 animals in T and for 5 animals in F. Similar letters represent no statistical differences (*P* > 0.05). *P* estimated by two-way ANOVA.

**Figure 3:**
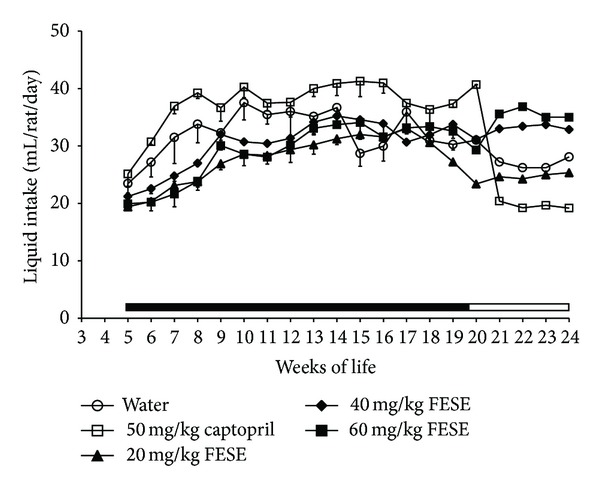
Liquid intake of spontaneously hypertensive rats. The animals drank different fluids from weaning until the 20th week of life (treatment period = T, indicated by a solid bar) and received therefore different daily treatments: tap water, 20 mg/kg/day *Fraxinus excelsior* L. seed extract (FESE), 40 mg/kg/day FESE, 60 mg/kg/day FESE, or 50 mg/kg/day captopril. All rats drank tap water from the 20th week of life until the 24th week of life (follow-up period = F, indicated by an open bar). Data are mean values ± SEM for 10 animals in T and for 5 animals in F. Similar letters represent no statistical differences (*P* > 0.05). *P* estimated by two-way ANOVA.

Development of hypertension was attenuated in the SHR groups treated with captopril or FESE. The antihypertensive effect was more accentuated in the group treated with captopril, and it was quite similar in all the FESE groups. Nevertheless, it was paradoxically more accentuated in the groups treated with 20 mg/kg/day or 40 mg/kg/day of FESE than in the group treated with the highest dose of this extract (60 mg/kg/day) (Figure 1). In addition, food intake increased somewhat in the FESE groups, and all the rats treated with this extract showed an increase in body weight gain. On the contrary, the animals treated with captopril showed less body weight than the rats of the water group, even if the food intake in all these animals was similar (Figure 2). The arterial blood pressure increased in the treated SHR when the corresponding antihypertensive treatment was removed. Nevertheless, at 24 weeks of life, systolic blood pressure remained lower in the captopril group than in the other groups. Body weight of the FESE treated animals returned to control values along the follow-up period. Liquid intake was variable in all animals along the experimental period, but we could appreciate a clear increase in this variable in the captopril treated rats (Figure 3).

### 3.2. Plasma Antioxidant Capacity

**Figure 4:**
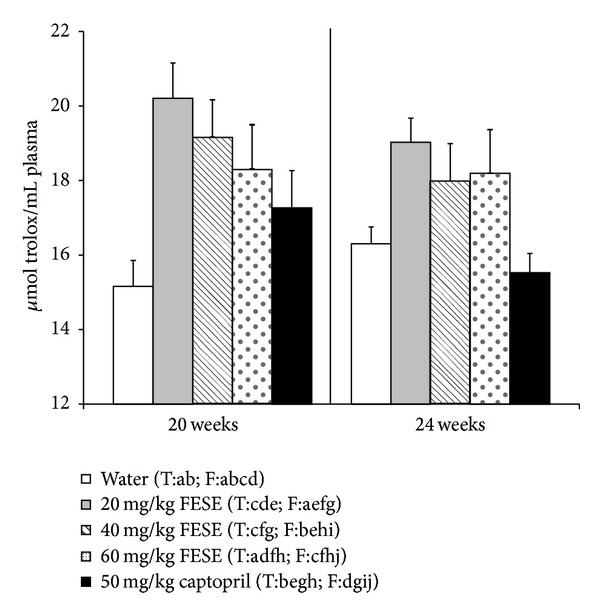
Histograms of the oxygen radical absorbance capacity (ORAC) of plasma samples (*μ*mol Trolox/mL plasma) from spontaneously hypertensive rats. The animals drank different fluids from weaning until the 20th week of life (treatment period = T) and received therefore different daily treatments: tap water, 20 mg/kg/day *Fraxinus excelsior* L. seed extract (FESE), 40 mg/kg/day FESE, 60 mg/kg/day FESE, or 50 mg/kg/day captopril. All rats drank tap water from the 20th week of life until the 24th week of life (follow-up period = F). Data are mean values ± SEM for 10 animals in T and for 5 animals in F. Similar letters represent no statistical differences (*P* > 0.05). *P* estimated by one-way ANOVA.

Plasma ORAC values in the 20-week old rats that had been long-term treated with 20 or 40 mg/kg/day FESE were significantly higher than the corresponding values in the control group. When these treatments were removed, plasma ORAC values returned to control values, and during the follow-up period (from the 20th to the 24th week of life) no significant differences were observed between the plasma ORAC values of these groups of rats and the corresponding values in the control rats that received tap water along all the experimental period. Long-term treatment with 50 mg/kg/day captopril did not modify plasma ORAC values in the rats and the animals that had been treated with this drug showed also similar ORAC values to the control animals along the follow-up period (Figure 4).

### 3.3. Plasma and Liver Malondialdehyde Levels

**Figure 5:**
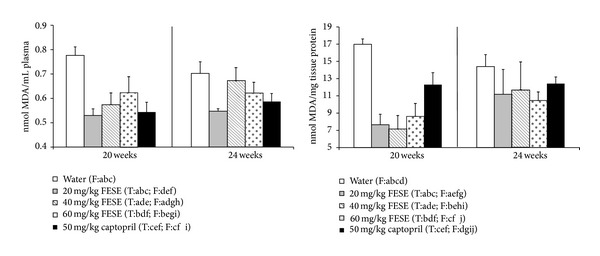
Malondialdehyde (MDA) equivalents in plasma and liver samples from spontaneously hypertensive rats. The animals drank different fluids from weaning until the 20th week of life (treatment period = T) and received therefore different daily treatments: tap water, 20 mg/kg/day *Fraxinus excelsior* L. seed extract (FESE), 40 mg/kg/day FESE, 60 mg/kg/day FESE, or 50 mg/kg/day captopril. All rats drank tap water from the 20th week of life until the 24th week of life (follow-up period = F). Data are mean values ± SEM for 10 animals in T and for 5 animals in F. Similar letters represent no statistical differences (*P* > 0.05). *P* estimated by one-way ANOVA.

Long-term treatment with all the used doses of FESE and also 50 mg/kg/day captopril significantly lowered plasma MDA equivalents in the SHR. Long-term treatment with 20 mg/kg/day FESE has the most accentuated effect, and when the corresponding treatments were removed, only MDA values of the rats that had been treated with this dose of the extract remained significantly lower when compared with the control group. In addition, long-term treatment with all the used doses of FESE and also 50 mg/kg/day captopril, significantly lowered liver MDA equivalents in the SHR when compared to control group. When the corresponding treatments were removed, all treated groups showed similar values compared to control group (Figure 5).

### 3.4. Aorta Vascular Relaxation

**Figure 6:**
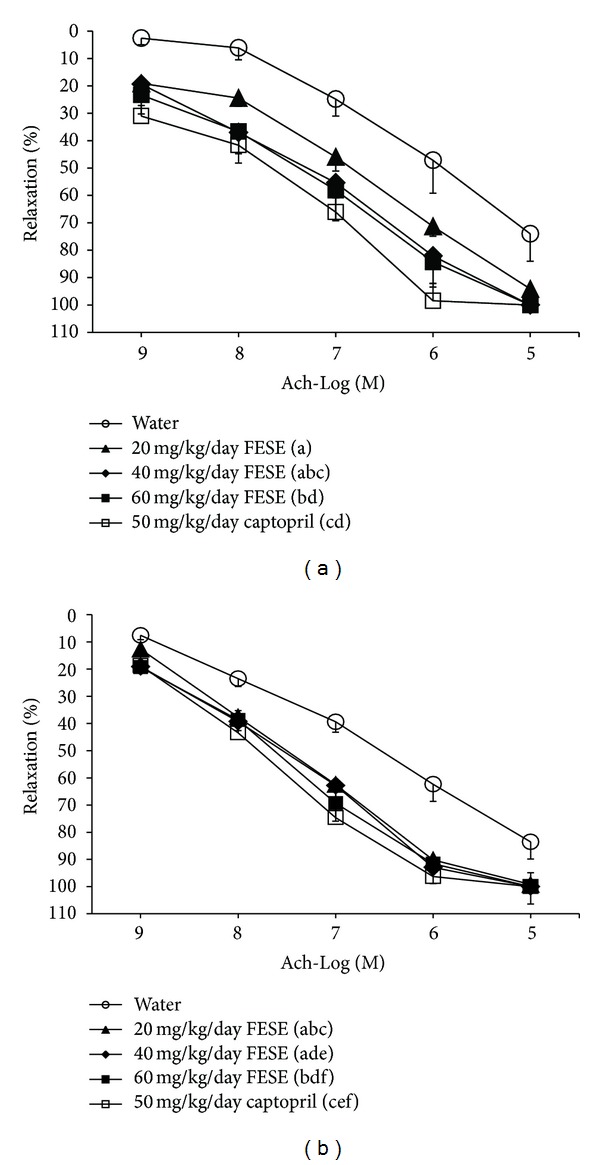
Cumulative concentration-response curves to acetylcholine (Ach) of aorta rings preconcentrated with 10^−5 ^M methoxamine from 20- (a) to 24-week-old (b) spontaneously hypertensive rats. The animals had received (from weaning until the 20th week of life) different daily treatments: tap water, 20 mg/kg/day *Fraxinus excelsior* L. seed extract (FESE), 40 mg/kg/day FESE, 60 mg/kg/day FESE, or 50 mg/kg/day captopril. All rats drank tap water from the 20th week of life until the 24th week of life (follow-up period). Data are mean values ± SEM for a minimum of 8 experiments. Similar letters represent no statistical differences (*P* > 0.05). *P* estimated by one-way ANOVA.

Acetylcholine relaxation improved in the aorta rings from the rats that had been treated with captopril or FESE. In particular, acetylcholine relaxation improved in the aorta rings from the rats that had been treated with captopril, but no significant differences were observed between the relaxations caused by this agonist in these preparations and the corresponding relaxations in aorta rings from the groups that had been treated with 40 mg/kg/day or 60 mg/kg/day of FESE. In fact, acetylcholine relaxation improved more in the aorta rings from the rats that had been treated with these doses of FESE than in the aorta preparations obtained from the 20 mg/kg/day FESE treated animals (Figure 6).

After the follow-up period, acetylcholine relaxation was also more marked in the aorta rings from the rats that had been treated with captopril or FESE than in the aorta rings from the rats that drank water along all the experimental period. No differences were observed between the different treated groups.

## 4. Discussion

In a previous study, we demonstrated the short-term antihypertensive effect of FESE in SHR. Nevertheless, this study clearly demonstrated that this extract can also control arterial blood pressure in these animals when it is long-term administered. SHR are considered nowadays one of the best experimental models to evaluate antihypertensive functional food ingredients [19]. The progression of hypertension in these animals is in fact similar to that in humans [20]. Several researchers, including our own research group, have reported that, before arterial blood pressure stabilizes in SHR, there is an initial period in the life of these animals in which this variable clearly increases [21–23]. After this period, the arterial blood pressure in the SHR remained constantly high. An initial gradual increase in SBP was in fact observed in the present study in the SHR of the control group, and we also noted the stabilized arterial blood pressure period in these animals. Both, FESE and captopril, attenuated the development of hypertension in the SHR and also clearly decreased their SBP in the period with stabilized arterial blood pressure values. Nevertheless, the decreases in SBP caused by all the used doses of FESE were always less accentuated than the decrease of this variable caused by 50 mg/kg/day captopril. In this context, it is important to notice that our research group characterized this dose of captopril as a very high dose, that caused the maximum effect when it was short-term administered to the SHR [13]. Captopril is in fact a well-known antihypertensive drug with clinical use in hypertensive patients and, as was expected, during the experimental period, the lowest values of SBP were observed in the group of rats treated with 50 mg/kg/day of this drug, but the antihypertensive effect of FESE was also maintained and could be clearly observed in the SHR stabilized arterial blood pressure period. The highest dose of FESE (60 mg/kg/day) caused even less effect than the other doses of this extract (20 mg/kg/day and 40 mg/kg/day), but neither in the short-term study with FESE, we obtained the most pronounced antihypertensive effect with the highest dose of this product [13]. Moreover, it is important to state that this paradox also happened with different polyphenols and polyphenol-rich compounds that we have evaluated in SHR [24].

After removing FESE treatments, arterial blood pressure rose gradually in the rats. In fact, from the 22nd week of life, SBP values were very similar in the rats that had drunk the solutions of FESE and those that had drunk water. Different researchers have similarly described a gradual rise in SBP when the intake of other antihypertensive food derived products was stopped in SHR [22, 25, 26]. We also observed an increase in the arterial blood pressure of the rats that had been treated with captopril when this treatment was removed. Nevertheless, the reversion was less noticeable, because this drug is a potent antihypertensive agent, and the rats that had drunk the solution of captopril had minor values of SBP than the other groups also during the follow-up period. Kost et al. (2000) [27] also reported a slight increase in arterial blood pressure in SHR following the withdrawal of captopril treatment.

SHR receiving captopril gained weight at a lower rate than the other animals even if the SHR treated with this drug did not show an increase in food intake. This finding is in agreement with previous studies showing that blockade of renin angiotensin system slows body weight gain in this rat strain [28]. It is nevertheless important to state that the SHR treated with FESE showed higher values of body weight than the control rats and slightly higher values of dry food intake. The mechanism implicated in this orexigenic effect of FESE is unknown and should be studied in the future. Nevertheless, liquid consumption increased markedly in the rats that drank the captopril solution, but this is not surprising because it has been clearly demonstrated that angiotensin converting enzyme inhibitors induce thirst and increase water intake and urine output in rats [29, 30].

The potential antioxidant therapy in hypertensive patients has been extensively studied [31]. Many plants with antihypertensive properties, that are having a marked impact on the food sector, are rich in phenolic antioxidant compounds. The relationship between natural phenol content in natural products and health is nevertheless still controversial, and there are problems to draw general conclusions regarding a health effect common to all phenolic compounds, as their structures are extremely diverse. In spite of these difficulties, it is important to have in mind that the secoiridoids nuzhenide and GI3 (in particular GI3), which are present in FESE, act as antioxidants [32] and have proved to have preventive effects on oxidative stress in rats [33]. Moreover, our research group has already demonstrated that the acute administration of FESE improves arterial blood pressure and the antioxidant state in SHR [13]. There exist a large number of methodologies aimed at evaluating the antioxidant capacity of complex mixtures [34]. ORAC type methodologies stand among the most employed ones to measure the total antioxidant potency of foods and nutritional supplements. In addition, MDA is a biomarker that enabled us to estimate lipid peroxidation in the rats. In this study, we have clearly demonstrated that the administration of 20 mg/kg/day or 40 mg/kg/day of FESE, the most effective antihypertensive doses of this extract, significantly increased plasma ORAC values in the SHR and significantly decreased plasma and liver MDA levels in these animals. Captopril failed nevertheless to increase the total antioxidant capacity of the SHR plasma samples, even if this drug enhances the antioxidant capacity in hypertensive patients [35] and has been postulated as a free radical scavenger [36, 37]. Moreover, FESE caused a more marked decrease in liver MDA than captopril in the SHR. The effects of FESE on the ORAC and MDA values did not totally disappear after removing the treatment. In particular, the plasma MDA values of the 24-week-old rats that had been treated with 20 mg/kg/day FESE remain significantly lower than those of the control rats of the same age. We should therefore assume that the antioxidant properties of FESE could justify, at least in part, the antihypertensive effects of this extract.

In this study, we could also assume that FESE improves endothelial function, since the relaxation to acetylcholine increased in the aorta rings from the SHR that had been treated with FESE as compared to the control group. The improvement was dose-dependent and was still observed in the aorta from the rats after the follow-up period. Nevertheless, the relaxation to acetylcholine increased always more in the aorta from the captopril positive control group than in the aorta from the FESE groups. These results suggest that the effect on endothelial function could also justify in part the antihypertensive effect of FESE.

In summary, long-term intake of FESE improves arterial blood pressure, oxidative stress, and endothelial function in SHR. The slight and maintained effect produced by FESE in the SHR suggests that this extract could be used as a functional food ingredient with potential therapeutic benefits in the prevention and treatment of hypertension and other associated disorders. Nevertheless, we are aware that before any clinical use of FESE, it would be necessary to carry out clinical studies to demonstrate its long-term antihypertensive efficiency in humans. Having in mind the results presented in this study, the possible orexigenic effect of FESE should also be studied in the future.
